# Cryogelation reactions and cryogels: principles and challenges

**DOI:** 10.55730/1300-0527.3586

**Published:** 2023-06-10

**Authors:** Oğuz OKAY

**Affiliations:** Department of Chemistry, Istanbul Technical University, İstanbul, Turkiye

**Keywords:** Cryogels, cryogelation reactions, squeezability, mechanical anisotropy, organohydrogels, shape-memory

## Abstract

Cryogelation is a powerful technique for producing macroporous hydrogels called cryogels. Although cryogelation reactions and cryogels were discovered more than 70 years ago, they attracted significant interest only in the last 20 years mainly due to their extraordinary properties compared to the classical hydrogels such as a high toughness, almost complete squeezability, a mechanically stable porous structure with honeycomb arrangement, poroelasticity, and fast responsivity against external stimuli. In this mini review, general properties of cryogelation systems including the cryoconcentration phenomenon responsible for the unique properties of the cryogels are discussed. The squeezability and poroelasticity of cryogels comparable to those seen with articular cartilage are also discussed. Cryogelation reactions conducted within the pores of preformed cryogels and some novel cryogels with attractive properties are then discussed in the last section.

## 1. Introduction

A hydrogel is a network of cross-linked hydrophilic polymers swollen in water. Since the pioneering discovery of poly(2-hydroxyethyl methacrylate) hydrogels as contact lenses by Wichterle and Lim in 1960 [1], several types of hydrogels have been developed for various applications including superabsorbent polymers, contact lenses, sensors, actuators, soft robotics, etc. [2]. One disadvantage of these so-called first generation hydrogels is their poor mechanical performance limiting their load-bearing applications. This poor performance is mainly due to their chemically cross-linked network structure with low energy dissipation [3,4]. Thus, when an external force is applied to a hydrogel, the force cannot be dissipated and is therefore localized at the point of application, resulting in a rapid crack propagation and subsequent fracture of the entire hydrogel sample. To overcome this limitation, various mechanisms have been developed to create an effective energy dissipation in hydrogels [5,6]. For example, the double-network hydrogels developed by Gong consist of brittle and ductile network components [7]. The brittle network breaks under force by dissipating energy, while the ductile network holds the macroscopic sample together.

**Figure 1:**
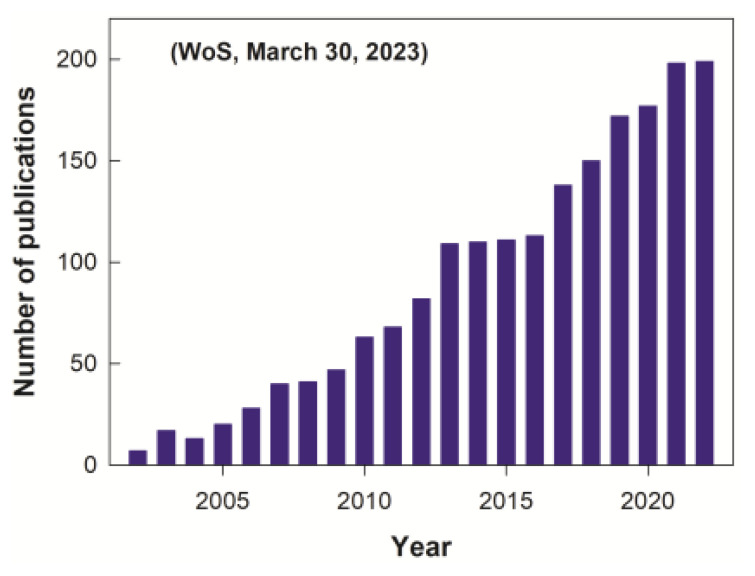
The number of publications with the key word “cryogels”. ISI Web of Knowledge database, March 30, 2023.

Cryogelation is a simple strategy for preparing mechanically strong macroporous hydrogels called cryogels [8–13]. Although cryogels were discovered more than 70 years ago, they attracted significant interest only in the last 20 years. This can be seen in the growing number of publications on cryogels during this time scale (Figure 1). The interest on cryogels is mainly due to their extraordinary properties compared to the classical hydrogels such as a high toughness, almost complete squeezability, a mechanically stable porous structure with honeycomb arrangement, poroelasticity, and fast responsivity against external stimuli.

A legend says that the history of cryogels is traced back to the time of the Soviet famine, a major famine in the Soviet Union that lasted from 1946 to 1948. During the famine, fish was transported by trains to Siberia, but some of the fish spoiled along the way. Stalin instructed researchers to find out how the fish could spoil at such low temperatures. Studies have shown that enzymes act to cause fish spoilage even at very low temperatures. However, the first real empirical studies on cryogelation systems were performed in 1970s at the A.N. Nesmeyanov Institute of Organoelement Compounds (IOEC) of the Russian Academy of Sciences [14]. The research was initially concentrated on food-type biopolymers and carried out within the framework of programs dealing with the chemistry and physical/colloid chemistry of food substances [14]. In fact, the research in this direction was triggered by an accidental discovery of a researcher, who placed a freshly extracted paste of Antarctic krill proteins in a freezer for the weekend so that the protein would not spoil. When the paste vessel thawed on Monday, there was a structured elastic gel-like product, i.e. a “protein cryogel” that took the form of this vessel.^1^ Vladimir I. Lozinsky, now the director of the Laboratory of Cryochemistry of (Bio)Polymers of IOEC, joined this field of research in 1980 in order to use synthetic models to understand the mechanisms of processes occurring in frozen polymeric systems [9,10]. He is the pioneer in the field of cryogenic processes and also made significant advances in the experimental aspects of a wide range of cryogels. Therefore, he can be considered the founder of cryogels based on synthetic and biological polymers. Cryogels and cryogenic processes have also attracted interest from scientists in Türkiye; in addition to my research group in Istanbul Technical University, research groups of Adil Denizli, Hacettepe University, and Nurettin Sahiner, Çanakkale Onsekiz Mart University, contributed significantly on the preparation of novel cryogels and their various applications.

In this mini review, I will first present general properties of cryogelation systems including the cryoconcentration phenomenon, the squeezability, and poroelasticity of cryogels. Some novel cryogelation techniques and cryogels with attractive properties are then discussed in the last section.

## 2. General properties of cryogelation systems

The major method for making cryogels involves freezing aqueous solutions of linear polymers or monomers and then cross-linking them. Unlike the usual gelation reactions carried out at or above the room temperature, cryogelation reactions are conducted 10 to 20 °C below the freezing point of the reaction system [11]. Under these cryogenic conditions, most of the water molecules are frozen, while a minute amount of them remain unfrozen due to the accumulated monomers or polymers. Thus, the polymerization and/or cross-linking reactions take place in the unfrozen regions of the apparently frozen system while the 3D network of ice crystals acts as a template for the pores.

**Figure 2:**
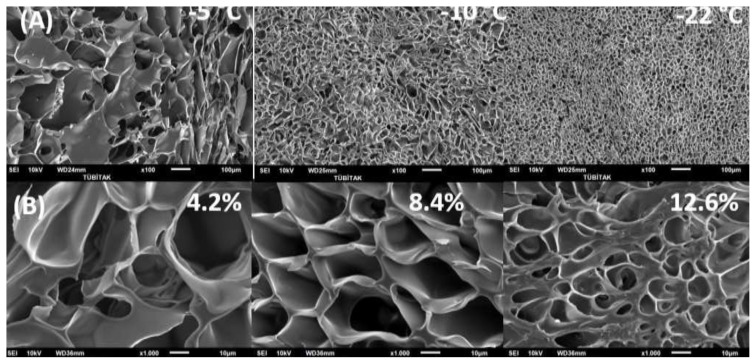
SEM images of silk fibroin (SF) cryogel scaffolds formed at various temperatures (A) and SF concentrations (B). Scaling bars are 100 (A) and 10 μm (B). (From [15] with permission from the American Chemical Society.)

The size of ice crystals and hence, the pore size of cryogels during cryogelation can be controlled by adjusting various experimental parameters including the freezing temperature and freezing rate of the cryogelation system. Lowering the cryogelation temperature or increasing the cooling rate can lead to smaller pores due to the formation of a larger number of small solvent crystals. This trend is seen in Figure 2A showing SEM images of silk fibroin (SF) cryogels formed at various temperatures [15]. The size and the wall thickness of the pores can also be influenced by the amount of monomers or polymers used during the cryogelation. Increasing monomer or polymer concentration generally leads to smaller pores and thicker pore walls [15–18]. For instance, the average pore size of SF cryogels prepared at −18 °C decreases from 33 ± 10 μm to 10 ± 3 μm as the amount of SF is increased from 4.2 to 12.6 wt% (Figure 2B) [15]. However, there are some exceptions to this trend, such as with polyelectrolyte cryogels formed at low monomer concentrations [19]. The relationship between monomer (or polymer) concentration and pore size is thought to be related to the number of unfrozen microregions during cryogelation, which increases as the concentration of solute is increased. Moreover, increasing the charge density of the network chains also leads to smaller pores [11].

**Figure 3:**
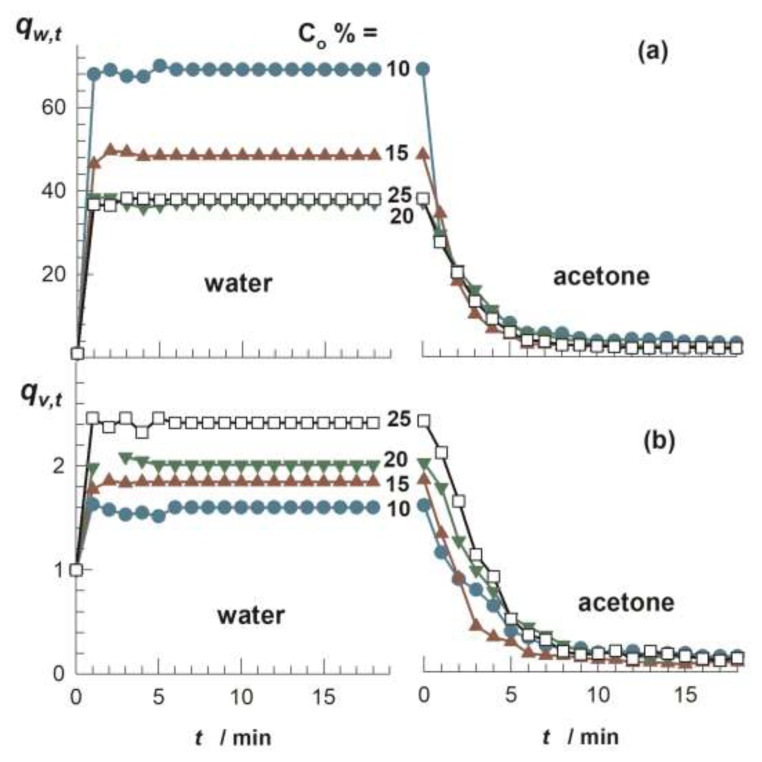
(a, b): *q_w,t_* (a) and *q_v,t_* (b) of PAMPS cryogels shown as a function of the contact time *t*. The cryogels are first immersed in water and then in acetone. AMPS concentration *C*_o_ is indicated. Experimental details are given in ref. 20. (From [20] with permission from Elsevier Ltd.)

Cryogels exhibit superfast responsivity against the external stimuli because of their interconnected 3D porous structure and elastic pore walls [17]. For instance, Figures 3a and 3b shows the weight *q_w,t_* and volume swelling ratios *q_v,t_*, respectively, for a cryogel specimen based on poly(2-acrylamido-2-methyl-1-propanesulfonic acid) (PAMPS) plotted against the contact time with solvent, which is first water and then acetone [20]. In contrast to the conventional PAMPS hydrogels requiring days to reach their swelling equilibrium in water, PAMPS cryogel attains its equilibrium state before the first measurement time of 1 min. Moreover, immersing the water-swollen cryogel in acetone, a poor solvent, it attains the equilibrium collapsed state within 5 min. It was shown that the swelling and deswelling cycles of PAMPS cryogels formed at various monomer concentrations are completely reversible [20]. Figures 3a and 3b also shows that the equilibrium weight swelling ratio *q_w_* of cryogels is much higher than their equilibrium volume swelling ratio *q_v_*. This is due to the fact that the volume swelling occurs due to solvation of the pore walls while the weight swelling also includes the solvent filling the pores of the cryogels [21]. As a consequence, *q_w_* is larger than *q_v_* for the cryogels, and the difference between them is proportional to their porosity.

Cryoconcentration phenomenon under cryogenic conditions is the basic characteristics of the cryogelation reactions, and determines cryogel properties. In the following sections, this phenomenon and unique characteristics of cryogels including squeezability and poroelasticity will be discussed.

### 2.1. Cryoconcentration

Cryoconcentration is a phenomenon observed during cryogelation, where the concentration of monomer (or polymer) increases in the unfrozen phase of the reaction system due to the separation of ice crystals. Thus, the cryoconcentration causes the monomer concentration to increase significantly in the unfrozen microchannels compared to its nominal concentration. For instance, in the copolymerization of N,N-dimethylacrylamide (DMAA) and poly(ethylene glycol) diacrylate in aqueous solutions, the local concentration of DMAA in the unfrozen zones was found to be 32.6 and 45.5 wt% at a cryogelation temperature of −10 and −20 °C, respectively, as compared to its initial (nominal) concentration of 6 wt% [18]. As a consequence, the reduction in the reaction rate caused by the low temperature is compensated for by the increase in the monomer concentration of the cryoconcentrated solution. Moreover, after cryogelation, the cryoconcentrated solution turns into a highly concentrated polymer hydrogel surrounding the ice domains. The cryogels can thus be produced at significantly lower nominal concentrations than traditional hydrogels. For instance, the minimum monomer concentration for the formation of a cross-linked polymer network during the free-radical cross-linking copolymerization of 2-acrylamido-2-methylpropane sulfonic acid (AMPS) and N,N’-methylenebisacrylamide (BAAm) decreases from 5 to 0.1 wt% when the gelation temperature is reduced from 25 to −22 °C [11].

**Figure 4:**
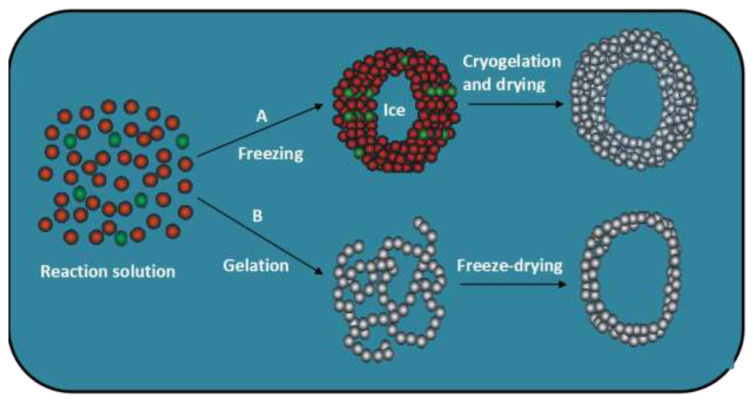
Formation of macroporous polymer scaffolds via two routes: (A) cryogelation and drying, (B) conventional gelation and freeze-drying. The monomers, cross-linkers, and polymer segments are presented by red, green, and gray circles, respectively. (From [11] with permission from Springer Nature.)

It should be mentioned that the cryogelation significantly differs from conventional gelation, where gelation occurs by the formation of intermolecular bonds in a homogeneous solution. During cryogelation, the freezing process occurs uniformly, allowing the formation of an interconnected network of ice crystals that serve as a template for the pores, together with a highly concentrated monomer solution (Figure 4A) [11]. This network of ice crystals also prevents the formation of large ice crystals that can damage the gel network. Subsequent polymerization and cross-linking reactions in the unfrozen domains of the cryogelation systems result in the formation of a dense polymer hydrogel acting as thick pore walls. Freeze-drying of an already formed hydrogel, on the other hand, does not lead to materials with cryogel properties because the gel is already formed and the cryoconcentration of the reaction constituents did not occur before the onset of gelation reactions (Figure 4B) [11].

**Figure 5:**
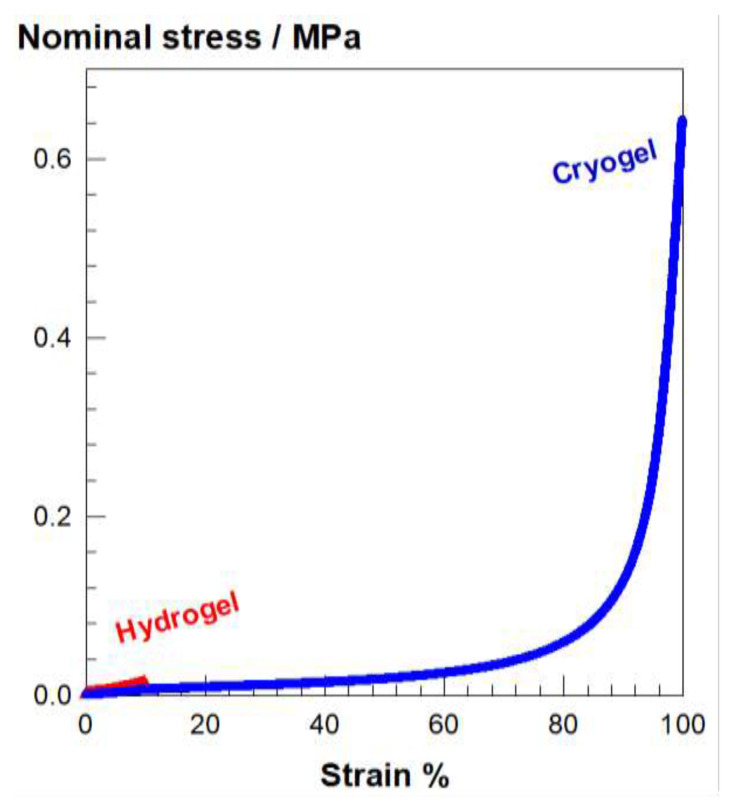
Stress-strain curves of SF-based hydrogel and cryogel specimens formed under identical conditions except the gelation temperature which is 50 and −18 °C, respectively. SF concentration = 4.2 wt%.

The cryoconcentration phenomenon significantly improves the mechanical properties of cryogels. To highlight this effect, two aqueous silk fibroin (SF) solutions at the same concentration (4.2 wt%) was cross-linked using 1,4-butanediol diglycidyl ether (BDDE) in the presence of N,N,N′,N′-tetramethylethylenediamine (TEMED) catalyst [15]. All the reaction parameters were kept constant except that the gelation temperature was taken as 50 and −18 °C to obtain SF hydrogel and cryogel, respectively. Their compressive stress-strain curves shown in Figure 5 reveal that the SF hydrogel ruptures at around 10% compression while the corresponding SF cryogel sustains up to almost compression without any failure. Moreover, the fracture stress of the cryogel is 70 times higher than that of the hydrogel (640 vs 9 kPa).

**Figure 6:**
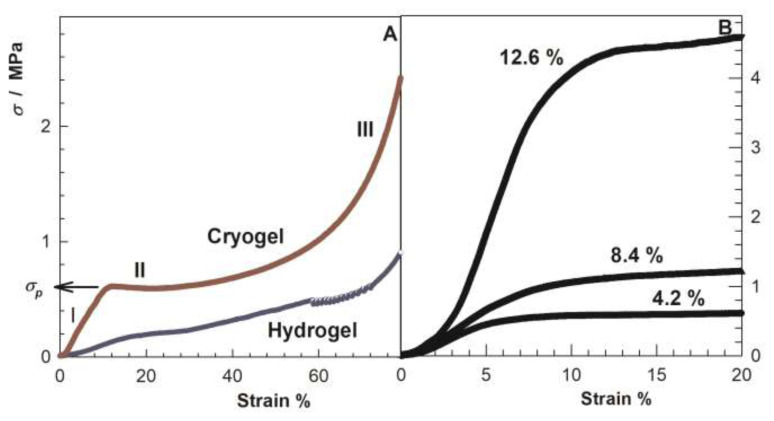
(A) Comparison of stress–strain curves of cryogel and hydrogel scaffolds prepared at −18 and 50 °C, respectively. (B): Stress-strain curves of cryogel scaffolds formed at −18 °C and at different SF concentrations as indicated. Experimental details are given in ref. 15. (From [15] with permission from the American Chemical Society.)

The extraordinary mechanical performance of cryogels compared to hydrogels is due to the cryoconcentration effect providing formation of a mechanically stable porous structure. By studying the stress-strain behavior of cryogels, one may gain insight into the stability of pore structure of the cryogels. This information can be used to design and optimize cryogels for specific applications, such as tissue engineering, drug delivery, and energy storage. The general trend is that the initial regime of the stress-strain curves is linear indicating that the pores remain mechanically stable (Figure 6A) [15]. However, as the cryogel is further compressed, the pores begin to deform due to the buckling of pore walls, which makes it easier to compress the cryogel. This leads to the appearance of a plateau regime in stress-strain curves.

The critical stress for this plateau regime, which is denoted by *σ_P_* in Figure 6A, is an indicator for the mechanical stability of the cryogels’ porous structure. The higher the *σ_P_*, the higher the mechanical stability of the pore structure. As seen in Figure 6B, increasing SF concentration at cryogelation also increases *σ_P_* and hence the pore stability due to the formation of thicker pore walls [15]. Moreover, the abrupt rise in the stress-strain curve in the third regime suggests the compression of the nearly nonporous polymer network (Figure 6A). In contrast to the cryogels, such distinct regimes are not seen in the stress-strain curves of hydrogels because of the absence of cryoconcentration.

### 2.2. Squeezability and poroelasticity

**Figure 7:**
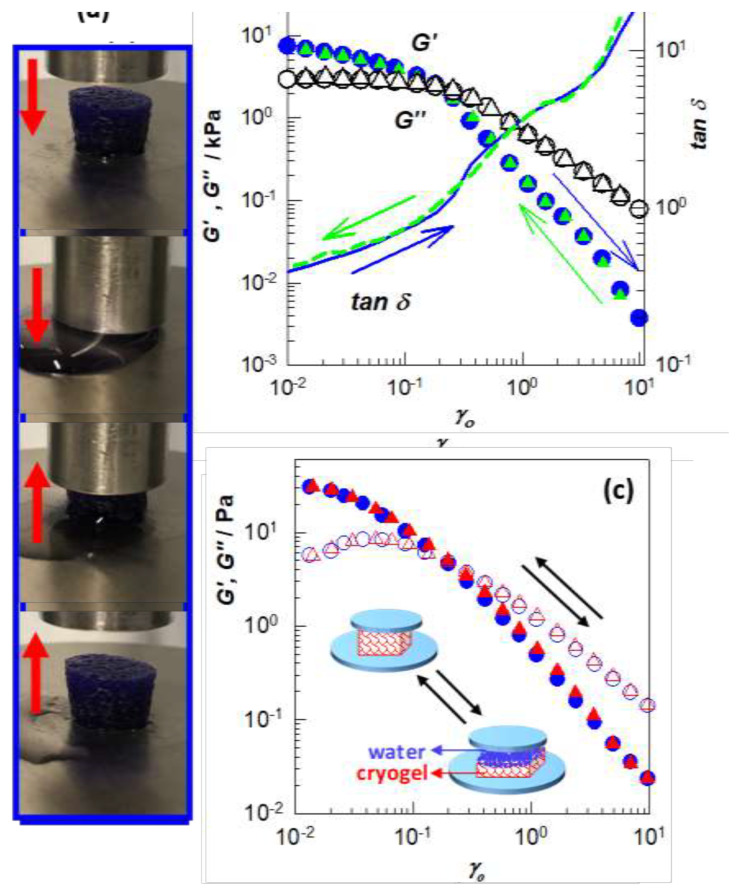
(a): Images of a HA cryogel specimen during loading, followed by unloading, as shown by the down and up arrows, respectively [22]. (b, c) Strain-sweep results of cryogels based on HA (b) [22], and SF (c) [23]. Experimental details are given in ref. 22 and 23. *G’*, *G”*, and tan δ are shown by the filled symbols, open symbols, and lines, respectively. The circles and triangles represent the data of the up and down strain sweep tests. The inset in (c) shows a cartoon presenting formation of a water film between the upper plate of the rheometer and the cryogel phase and at high strains. (From [22, 23] with permission from Elsevier Ltd.)

Cryogels formed under certain conditions also exhibit total squeezability and flow-dependent viscoelasticity called poroelasticity. For instance, Figure 7a illustrates the squeezability of a cryogel specimen-based hyaluronic acid (HA) [22]. The cryogel can completely be compressed under force without any damage during which water is squeezed out of the pores, but reversibly, when the force is removed, the cryogel again absorbs water to recover its original shape. Due to water flowing into and out of the pores, much like water squeezing out of a sponge, HA cryogels also display reversible strain-dependent apparent gel-to-sol transition behavior [22].

This behavior was demonstrated by cyclic strain-sweep experiments conducted on HA cryogels between 1% and 100% strains (γ_o_). Figure 7b depicts *G’*, loss modulus *G”*, and loss factor tan δ of a cryogel sample under cyclic strain changes [22]. Circles and triangles, respectively, represent the outcomes of the up and down strain sweep tests. It is seen that at ≥20% strain, *G”* dominates over *G’*; hence, the cryogel behaves as a low density liquid, while when the strain was returned to 1%, the cryogel regained its original viscoelastic characteristics, demonstrating the reversibility of the solid to liquid-like transition. Similar results were also reported for SF cryogels (Figure 7c) [23], and how water moving in and out of the pores affects the viscoelastic response of cryogels is demonstrated. It is important to note that this gel-to-sol transition is only an apparent transition because the gel network is still intact under stress.

This flow-dependent viscoelasticity of cryogels, also referred to as poroelasticity, is comparable to that seen with articular cartilage, a load-bearing and low-friction soft tissue that has been providing the joint with essential biomechanical functions, such as shock absorption, load bearing, and wear resistance [24–27]. As observed in HA and SF cryogels, when the cartilage is compressed, the liquid within the tissue escapes via the pores, causing significant frictional resistance and, in turn, frictional energy dissipation, which is what causes the viscoelastic behavior of the articular cartilage. The apparent gel-to-sol transition behavior of both HA and SF cryogels is also of significant interest in biomaterial and biological applications because it serves as a self-defense mechanism by preventing the destruction of their networks under high strains.

## 3. Novel cryogelation techniques and cryogels

In this section, recent advances in the field of cryogelation and cryogels are summarized. These include cryogels with mechanical anisotropy, cryogelation reactions confined within the pores of cryogels, and organohydrogels mimicking biological systems.

### 3.1. Cryogels with mechanical anisotropy

Many biological tissues have anisotropic hierarchical morphologies providing them exceptional mechanical capabilities that cannot be mimicked by synthetic materials. This unique morphology of biological tissues including intervertebral disc [28], cornea [29], muscles [30], cartilage [31], and tendon [32] was not observed in synthetic hydrogels with isotropic morphologies. A significant challenge in gel science is thus to produce mechanically robust macroporous hydrogels with anisotropic characteristics. Several methods, including directed freezing [33,34], stress-induced orientation [35], and self-assembly [36] have been designed to produce hydrogels with anisotropic microstructure. The modulus anisotropy of hydrogels containing oriented clay nanotubes or fibrils is 3.0 and 7.0, respectively [37,38]. Polyvinyl alcohol and double-network hydrogels were similarly shown to exhibit modulus anisotropies between 2 and 4 [39,40]. The modulus anisotropy of polyacrylic acid hydrogels produced by directional ice-crystal growth and subsequent polymerization is 10.4 [41]. Nonetheless, all of these hydrogels have weak mechanical properties; specifically, their moduli measured in the directions parallel and perpendicular to the orientation direction of the materials are 80.5 and 7.7 kPa, respectively. To maintain their integrity, anisotropic scaffolds used in tissue engineering applications should have a modulus of the order of MPa.

**Figure 8:**
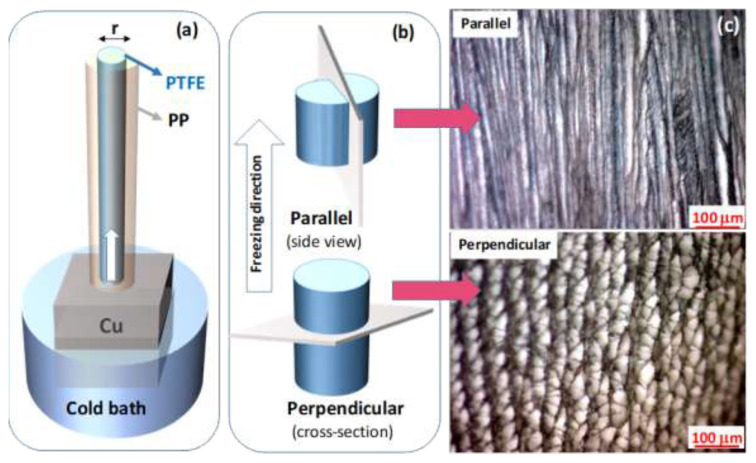
(a) Experimental setup for synthesis of SF cryogels with an anisotropic pore structure. (b) Freezing direction of aqueous SF solution in the cryogelation reactor. (c) Optical images of the cryogels taken in directions parallel and vertical to the freezing direction. SF = 4.2 wt %. (From [23] with permission from Elsevier Ltd.)

**Figure 9:**
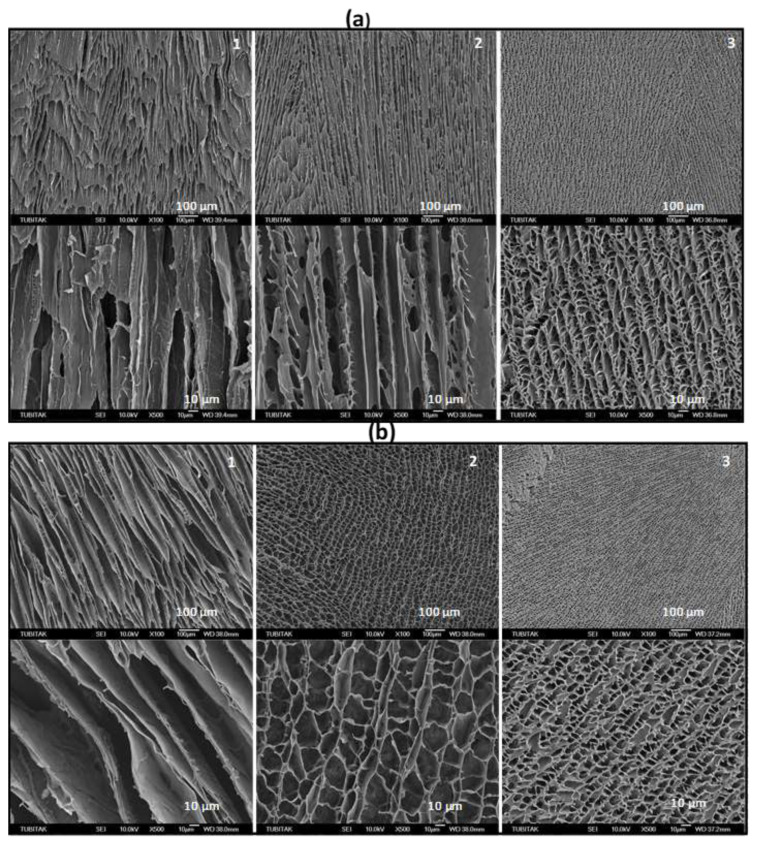
SEM photos of dry state cryogels prepared at 2.1 (1), 4.2 (2), and 16.7 wt. SF % (3). They were taken parallel (a) and perpendicular to the freezing direction (b). (From [23] with permission from Elsevier Ltd.)

Recently, a simple technique for generating mechanically robust SF scaffolds with a high degree of mechanical anisotropy similar to tendon was reported by directional freezing of a cryogelation system [23,42]. A reactor with a copper bottom plate and a cylindrical polytetrafluoroethylene (PTFE) mold with a thermal conductivity ratio of 1600 were designed to produce these cryogels (Figure 8a). The cylindrical PTFE mold, which is located outside of the cold bath, was filled with aqueous solutions of SF in varying percentages together with BDDE and TEMED as a cross-linker and pH regulator, respectively. The copper bottom plate was submerged in a cold bath at −30 or −196 °C. The unidirectional frozen fibroin solution was then subjected to cryogelation reaction at −18 °C during which a conformational change in fibroin from random coil to β-sheet structure and hence cryogelation occurred [42]. Using this procedure, a series of mechanically strong anisotropic SF scaffolds were obtained. In order to characterize the mechanical anisotropy of the cryogels, they were subjected to uniaxial compression tests along parallel and perpendicular to the freezing direction (Figure 8b). Figure 8c presenting the optical images of the cryogels reveals the alignment of the pores parallel to the freezing direction. Figures 9a and 9b shows SEM images of SF cryogels prepared at three different SF concentrations, namely, 2.1, 4.2, and 16.7 wt% (from left to right) [23]. The images were taken parallel (Figure 9a) and perpendicular to the freezing direction (Figure 9b). At 2.1 wt% SF (left panel), the pores partially collapse while at higher SF concentrations, the cryogels exhibit a channel-like porous structure consisting of several hundred μm long, aligned fibroin layers interconnected by vertically oriented branches. In accord with Figure 2, increasing concentration of SF decreases the size of the pores and the spacing between the fibroin layers.

**Figure 10:**
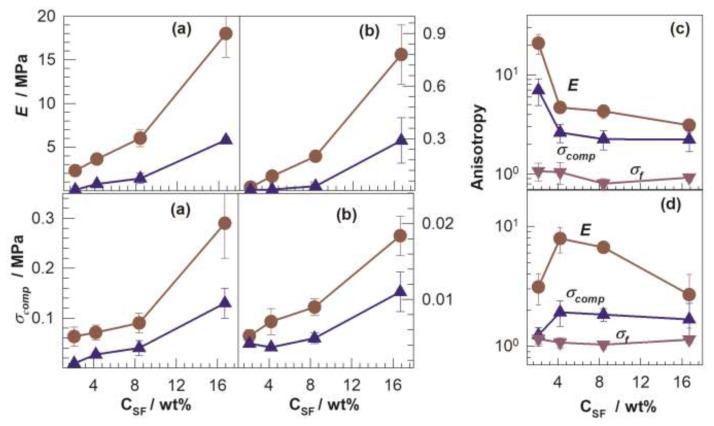
(a, b): The mechanical parameters *E* and *σ*_comp_, of cryogels in their dry (a) and swollen states (b) plotted against SF concentration. The circles and triangles represent the data measured in parallel and perpendicular to the freezing direction. (c, d): Anisotropy of the cryogels with respect to *E*, *σ*_comp_, and *σ*_f_ in their dry (c) and swollen states (d) for various SF concentration. (From [23] with permission from Elsevier Ltd.)

Mechanical tests carried out along parallel and perpendicular to the freezing direction indicate a higher stiffness (modulus) of the cryogels in the parallel directions. This behavior is also seen in Figures 10a and 10b presenting Young’s modulus *E* and fracture stress *σ*_comp_ of the cryogels in dry and swollen states, respectively, plotted against SF concentration [23]. The circles and triangles in the figures represent the data recorded along parallel and perpendicular to the freezing direction, respectively. Both *E* and *σ*_comp_ of the cryogels are higher in the parallel direction than the perpendicular one. The degree of anisotropy which is the ratio of a mechanical parameter measured parallel to perpendicular directions is shown in Figures 10c and 10d for cryogels in dry and swollen states, respectively, as a function of SF concentration. The cryogel formed at the lowest SF concentration of 2.1 wt% and in dry state exhibits the greatest modulus anisotropy yet recorded, 21 ± 5, i.e. the moduli of the cryogel measured parallel to and perpendicular to the freezing direction are 2.3 ± 0.5 and 0.11 ± 0.03 MPa, respectively. Concluding this section, the simple technique summarized above produces cryogels with a high degree of mechanical anisotropy similar to that of tendons. Such cryogels have potential applications in tissue engineering and regenerative medicine.

### 3.2. Cryogel micropores as the reaction loci for cryogelation reactions

**Figure 11:**
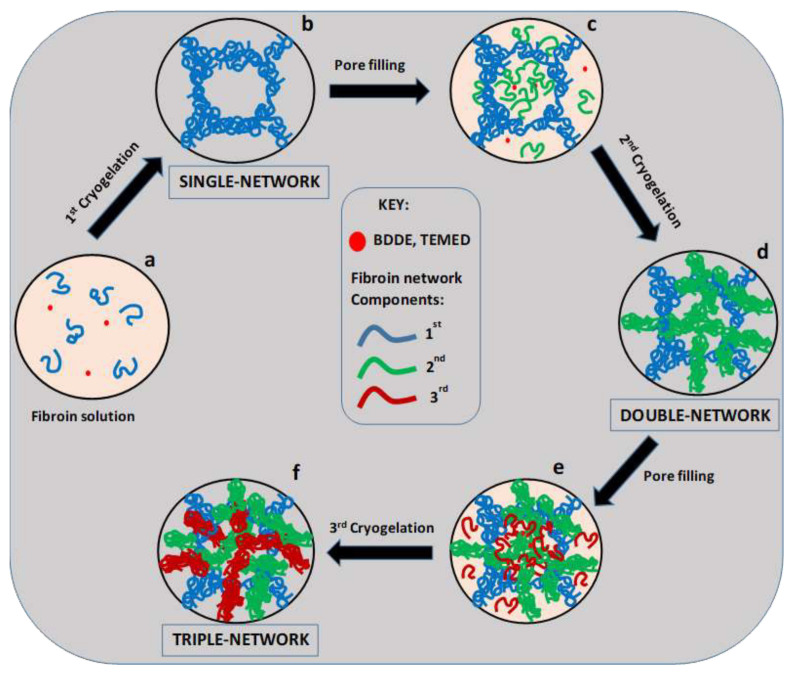
Cartoon presenting preparation of cryogels with SN, DN, and TN structures. (From [43] with permission from Elsevier Ltd.)

The cryogelation reactions can also be conducted inside the pores of a preformed cryogel scaffold to obtain multiple generation of pores [43]. Such scaffolds having multiple-sized pores are attractive materials in tissue engineering because large and small pores in the order of micrometers are suitable for cell migration and nutrient delivery, respectively [44–48]. Cryogels based on SF with multiple-sized pores were generated by immersing a cryogel scaffold in an aqueous SF solution containing BDDE and TEMED, followed by the cryogelation reactions to obtain cryogels with a double-network (DN) structure (Figures 11a–11d). By repeating this procedure, SF cryogel with a triple-network (TN) structure was obtained (Figures 11e and 11f). Thus, by successive pore-filling–cryogelation steps, SF cryogels with single-network (SN), double-network (DN), and triple-network (TN) structures with adjustable pore sizes could be obtained [43].

**Figure 12:**
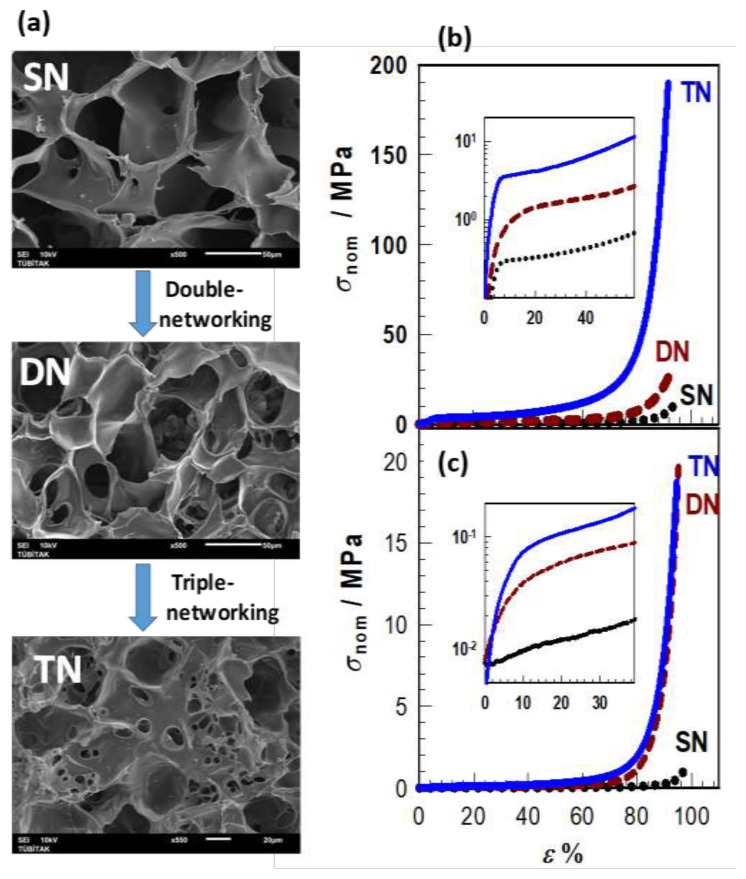
(a) SEM images of SN, DN, and TN scaffolds. Single-, double-, and triple-networking were conducted at SF concentrations of 4, 7, and 20 wt%, respectively. Scale bars = 20 μm (TN) and 50 μm (DN and SN). (b, c) Stress-strain curves of SN, DN, and TN cryogels in their dry (b) and swollen states (c). The insets show the same data up to 40%–50% strain in semi-logarithmic plot. (From [43] with permission from Elsevier Ltd.)

For instance, Figure 12a presents SEM images of a cryogel scaffold with TN structure and its DN and SN precursors. The single-, double-, and triple-networking were conducted at SF concentrations of 4, 7, and 20 wt%, respectively. The large pores in SN become smaller after double-networking due to the partial filling of the pores with the second network. In addition, a second generation of pores appears in DN scaffold with 8 ± 1 μm in diameter. Moreover, triple-networking of DN further decreases the diameter of the small pores to 2 ± 1 μm. It was also shown that the size of both small and large pores could be tuned by the relative contents of SF in the network components [43]. The mechanical performance of the cryogels significantly increased after multiple cryogelation reactions [43]. Figures 12b and 12c show stress-strain curves of dry (Figure 12b) and swollen cryogels with SN, DN, and TN structures. Both the fracture stress and plateau stress increase in the order of SN → DN → TN while fracture strain remains unchanged. At an SF concentration of above 25 wt%, TN cryogels exhibit a Young’s modulus in the range of 66–126 MPa, and can sustain 90% compressions under 87–240 MPa stresses. These values are the highest so far reported for fibroin scaffolds; hence, the scaffolds after multiple-networking have potential use in bone tissue engineering.

### 3.3. Organohydrogels

When compared to synthetic hydrogels, their biological counterparts like cells and tissues have complementary biphasic components with hydrophilic and lyophilic properties [49]. The coexistence of these opposing characteristics in the body provides some smart functions such as adaptive biomechanics and freezing tolerance. For instance, echinoderms like sea cucumbers and starfish that have malleable collagenous tissue may regulate their body mechanics by switching between a hard and a soft mode when attacked or at rest, respectively [50–52]. Moreover, Arctic wood frogs can also endure in cold and dry environments without freezing because of their antifreeze proteins comprising both hydrophilic and lyophilic fragments [53,54].

Hydrogels possessing both hydrophilic and lyophilic domains, called organohydrogels (OHGs), were recently reported by mimicking the biological systems. OHGs attract great interest for various application areas such as freeze-tolerant materials, signal transmission, sensors, soft robotics, and optical devices [55–58]. Dispersing a discontinuous organic (lyophilic) phase in a continuous aqueous phase to create an oil-in-water emulsion followed by polymerization is a commonly used technique to prepare an OHG [59]. A series of OHGs with freeze-tolerant properties were also prepared by adding a lyophilic phase to a freeze-dried poly(DMAA) hydrogel [60]. Similarly, antifreezing agents such as sorbitol, glycol, and glycerol were incorporated into a poly(acrylamide)/alginate hydrogel network to produce nonfreezable hydrogels [61].

**Figure 13:**
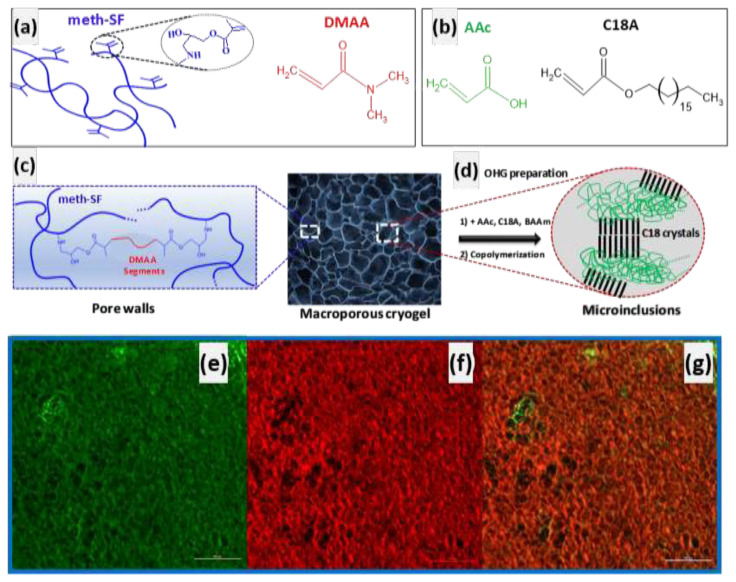
(a–c) Chemical structure of the components of the hydrophilic and hydrophobic phases of the OHG [62]. (d) The formation of semicrystalline poly(AAc-co-C18A) organogel microinclusions by filling the pores of the cryogel with AAc, C18A, and BAAm monomers, followed by polymerization in the presence of AIBN. (e–g) Confocal microscopy images of the OHG. FITC was used to color the cryogel scaffold green, and Nile red was used to color the poly(AAc-co-C18A) microinclusion. The images display the SF scaffold (e) and organogel phases (f), and the OHG (g). Scale bars indicate 100 μm. (From [62] with permission from the American Chemical Society.)

Recently, a new method was reported for creating adaptive OHGs with tunable viscoelasticity and mechanics [62]. The method involves using a hydrophilic cryogel scaffold as the continuous phase, and filling the scaffold’s pores with a hydrophilic monomer, crystallizable hydrophobic comonomer, chemical cross-linker, and an initiator, followed by free-radical polymerization. More specifically, an aqueous solution of methacrylated SF (meth-SF) was first subjected to cryogelation reactions at −18 °C to produce SF cryogel possessing 94% porosity and interconnected micrometer-sized pores (Figure 13). The cryogel was then submerged in an ethanolic solution of organogel precursors containing acrylic acid (AAc), n-octadecyl acrylate (C18A), N,N’-methylenebis(acrylamide), and a free-radical initiator. The free-radical polymerization within the pores of the cryogel results in adaptive OHGs consisting of SF scaffold containing semicrystalline poly(AAc-co-C18A) organogel microinclusions [62].

**Figure 14:**
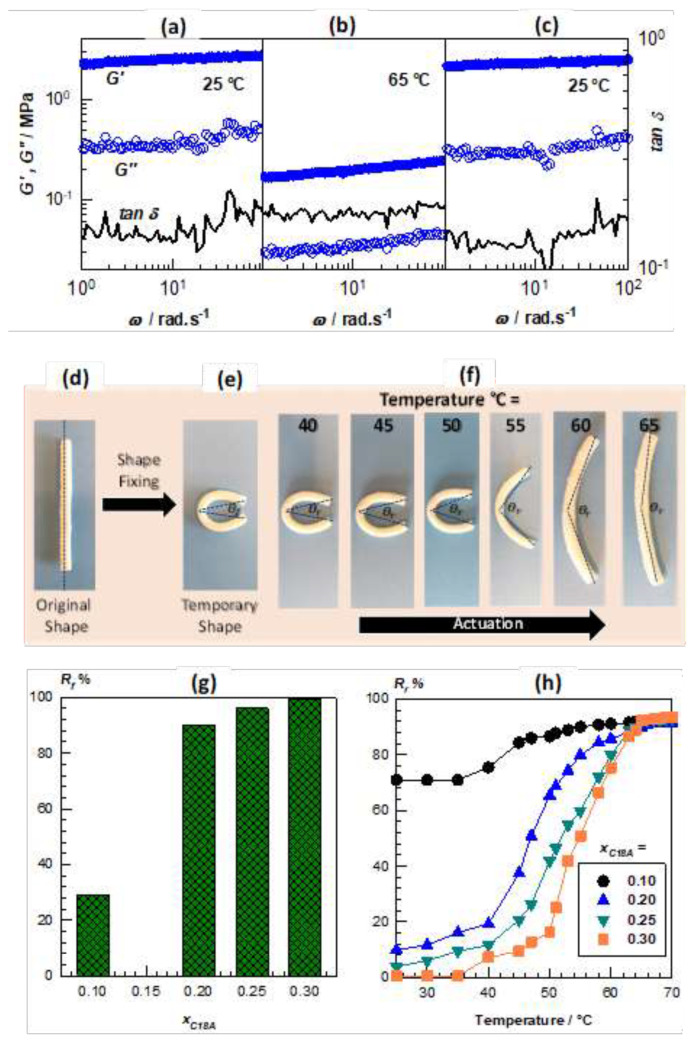
(a–c) Frequency (*w*) dependences of *G’*, *G”* and *tan* δ of an OHG sample at 25 (a) and 65 °C (b), and after cooling back to 25 °C (c). γ_o_ = 0.1%. (d–f) Images showing the shape-memory behavior of an OHG prepared at x_C18A_ = 0.30. (g, h) *R_f_* (g), and the temperature dependent *R_r_* (h) for OHGs prepared at various C18A contents x_C18A_. (From [62] with permission from the American Chemical Society.)

The melting temperature T_m_ and the degree of crystallinity of OHGs can be adjusted between 49 and 54 °C and 1.3%–13%, respectively, depending on the molar fraction x_C18A_ of the crystallizable C18A monomer units; hence, they have switchable mechanics and viscoelasticity in response to temperature changes between below and above T_m_. For instance, Figures 14a–14c show the frequency dependences of *G’*, *G”*, and *tan* δ of an OHG specimen prepared at x_C18A_ = 0.30. The frequency sweep tests were first carried out at 25 °C (Figure 14a), then after heating to 65 °C (Figure 14b), and finally after cooling back to 25 °C (Figure 14c). At 25 °C, i.e. below T_m_ of the crystalline domains, the OHG exhibits a modulus *G’* of around 2 MPa while it decreases to 0.2 MPa upon heating above T_m_. This decrease in *G’* is reversible, i.e. cooling the OHG specimen from 65 to 25 °C recovers its initial modulus *G’*, indicating that OHG has reversibly switchable viscoelasticity in response to a change in the temperature between below and above T_m_. The reversible hard-to-soft transition in the mechanical properties depending on temperature is a prerequisite for the shape-memory effect.

Indeed, all OHGs exhibited an effective temperature-induced shape-memory effect [62]. The photos in Figures 14d–14f demonstrate the shape-fixity and shape–recovery characteristics of an OHG specimen with x_C18A_ = 0.30. The permanent shape of the specimen is rod (Figure 14d). It is deformed to give a horseshoe-like temporary shape above T_m_ (65 °C) which is then fixed by cooling below T_m_ (Figure 14e). By raising the temperature above T_m_, the specimen recovers its original permanent shape (Figure 14f). The shape-memory behavior of OHGs is due to the coexistence of C18 crystals in the microinclusions and the cryogel matrix acting as switching segments and netpoints, respectively. Figure 14g demonstrates that the shape-fixity ratio *R_f_* dramatically rises as the C18A content (x_C18A_), i.e. the degree of crystallinity of OHG increases. *R_f_* increases from 29% to 99.5% with rising x_C18A_ and becomes complete at x_C18A_ = 0.30. Moreover, all OHGs at 70 °C show a shape-recovery ratio *R_r_* of greater than 92%, demonstrating their switchable mechanical characteristics in response to temperature (Figure 14h). To conclude, the strategy developed for creating OHGs with shape-memory function is suitable for a wide range of semicrystalline organogels and hydrophilic scaffolds and could be used in various applications, including electronics, aerospace, and tissue and biomedical engineering.

## 4. Conclusion

Cryogelation reactions and cryogels have attracted significant interest in the last 20 years due to their extraordinary properties compared to the classical hydrogels such as high toughness, almost complete squeezability, mechanically stable porous structure with honeycomb arrangement, poroelasticity, and fast responsivity against external stimuli. In this mini review, the principles of cryogelation systems and the general properties of cryogels are presented. I mainly focused on the cryoconcentration phenomenon, which is the accumulation of monomers or polymers in the unfrozen solution that occurs during ice formation. The polymerization and/or cross-linking reactions taking place in the cryoconcentrated solution produce an elastic hydrogel acting as the pore walls of the resulting cryogels. The combination of high mechanical strength, porosity, and responsiveness due to cryoconcentration makes cryogels promising materials for a variety of applications, including drug delivery, sensors, and tissue engineering. Another characteristic of biological cryogels based on HA and SF discussed in this review is their total squeezability and flow-dependent viscoelasticity called poroelasticity. The cryogels can completely be compressed under force without any damage during which water is squeezed out of the pores, but reversibly, when the force is removed, they again absorb water to recover their original shape. Due to water flowing into and out of the pores, much like water squeezing out of a sponge, they also display reversible strain-dependent apparent gel-to-sol transition behavior. This flow-dependent viscoelasticity of cryogels, also referred to as poroelasticity, is comparable to that seen with articular cartilage, a load-bearing and low-friction soft tissue that has been providing the joint with essential biomechanical functions, such as shock absorption, load bearing, and wear resistance. The poroelasticity of cryogels is of significant interest in biological and biomaterial applications because it serves as a self-defense mechanism by preventing the destruction of their networks under high strains. In recent years, various novel cryogelation techniques have been developed to produce cryogels with improved properties. For example, the use of the pores of the cryogel scaffolds as the reaction loci for cryogelation reactions produce multiple-network cryogels with large and small pores. Cryogels with aligned pores and anisotropic mechanical properties, and organohydrogels consisting of a hydrophilic cryogel and hydrophobic semicrystalline organogels are examples of recently developed cryogels with attractive properties. Novel cryogelation techniques and cryogels with extraordinary properties continue to be developed, opening up new possibilities for their use in various applications.

## Footnotes

1 Vladimir I. Lozinky, March 2023, private communication.
